# Supplementary material for: Transcriptomic Changes Associated with ERBB2 Overexpression in Colorectal Cancer Implicate a Potential Role of the Wnt Signaling Pathway in Tumorigenesis
Source: Cancers (Basel). 2022 Dec 26;15(1):130. doi: 10.3390/cancers15010130 (PMC9817785; doi:10.3390/cancers15010130)

Supplementary Data

Transcriptomic changes associated with ERBB2 overexpression in colorectal cancer implicates a potential role of Wnt signaling pathway in tumorigenesis

Eman A. Abdul Razzaq, Khuloud Bajbouj, Amal Bouzid, Noura Alkhayal, Rifat Hamoudi and Riyad Bendardaf

Supplementary Tables S5-S7

Supplementary Figure Legends S1-S7

Supplementary Figures S1-S7

Supplementary Table S5. Summary of the total activated cellular pathways identified by GSEA in *ERBB2*+ versus *ERBB2*- colorectal cancer (CRC) patients, CRC cell lines HCT116 and HT29, normal colon cell lines CCD33 and CCD841, and breast cancer (BC) patients ($p < 0.05$).

	**C2**	**C5-BP**	**C5-MF**	**C6**	**C7**
CRC Patients: 3 HER2- vs 11 HER2+	9	11	0	5	73
HCT116: 3 Ctrl EV vs 3 HER2+	6	3	0	0	6
HT29: 3 Ctrl EV vs 3 HER2+	21	24	2	0	42
CCD33 3 Ctrl EV vs 3 HER2+	0	0	0	0	0
CCD841 3 Ctrl EV vs 3 HER2+	2	0	4	0	0
BC Patients: 17 HER2+ vs 48 HER2-	908	1379	224	112	2017

Supplementary Table S6: Details of breast cancer datasets analyzed to determine correlation of *ERBB2*-mediated enrichment of pathways in breast and colon cancer patients. *N*, total number of breast cancer patients.

Data Set ID	Population	N	Comparison of Samples	
GSE29431	Caucasian/Spanish	21	13 HER2-	8 HER2+
GSE48391	Asian/Chinese	44	35 HER2-	9 HER2+
		**65**	**48 HER2-**	**17 HER2+**

Supplementary Table S7. The 26 common activated pathways among CRC and BC HER2+ versus HER2- patients obtained from Gene Set Enrichment Analysis. *ES*, enrichment score; *NES*, normalized ES; *NOM*, nominal; *Tag%*, the percentage of gene tags before (for positive ES) of after (for negative ES) the peak in the running enrichment score; *Gene %*, the percentage of genes in the gene list before (for positive ES) of after (for negative ES) the peak in the running enrichment score; *GO*, gene ontology.

Gene Sets	Class	SIZE	ES	NES	NOM p-val	Tag %	Gene %	Signal
GSE16385_UNTREATED_VS_12H_ROSIGLITAZONE_TREATED_MACROPHAGE_DN	C7	17	0.58757	1.8687	0	0.471	0.127	0.413
GSE25088_WT_VS_STAT6_KO_MACROPHAGE_ROSIGLITAZONE_AND_IL4_STIM_UP	C7	20	0.47997	1.6523	0.002299	0.7	0.426	0.405
GSE17721_PAM3CSK4_VS_CPG_16H_BMDC_DN	C7	20	0.45969	1.5947	0.005319	0.5	0.274	0.366
GSE9006_TYPE_1_DIABETES_AT_DX_VS_4MONTH_POST_DX_PBMC_DN	C7	25	0.48856	1.6076	0.006438	0.6	0.399	0.364
GSE39022_LN_VS_SPLEEN_DC_UP	C7	19	0.53179	1.6277	0.009546	0.632	0.317	0.435
GSE15659_NONSUPPRESSIVE_TCELL_VS_ACTIVATED_TREG_UP	C7	30	0.45884	1.7067	0.01013	0.533	0.248	0.406
GSE26351_WNT_VS_BMP_PATHWAY_STIM_HEMATOPOIETIC_PROGENITORS_UP	C7	27	0.46925	1.579	0.01295	0.63	0.396	0.384
GSE15624_CTRL_VS_6H_HALOFUGINONE_TREATED_CD4_TCELL_DN	C7	26	0.49551	1.4309	0.01431	0.538	0.304	0.378
GSE7509_UNSTIM_VS_FCGRIIB_STIM_DC_UP	C7	22	0.48209	1.5959	0.02103	0.545	0.28	0.396
GSE37534_UNTREATED_VS_ROSIGLITAZONE_TREATED_CD4_TCELL_PPARG1_AND_FOXP3_TRASDUCED_UP	C7	34	0.36357	1.4181	0.02529	0.765	0.548	0.35
GSE10147_IL3_VS_IL3_AND_CPG_STIM_PDC_DN	C7	18	0.53648	1.5054	0.03178	0.556	0.241	0.424
GSE5542_UNTREATED_VS_IFNG_TREATED_EPITHELIAL_CELLS_24H_UP	C7	24	0.56425	1.5257	0.03272	0.667	0.33	0.451
GSE3982_MAST_CELL_VS_NKCELL_DN	C7	25	0.42148	1.4406	0.035	0.56	0.332	0.378
GSE5589_LPS_VS_LPS_AND_IL10_STIM_MACROPHAGE_45MIN_UP	C7	21	0.49249	1.4864	0.03846	0.619	0.337	0.414
GSE5542_UNTREATED_VS_IFNA_AND_IFNG_TREATED_EPITHELIAL_CELLS_6H_UP	C7	25	0.43579	1.444	0.04065	0.44	0.22	0.346
GSE34156_UNTREATED_VS_24H_NOD2_AND_TLR1_TLR2_LIGAND_TREATED_MONOCYTE_DN	C7	25	0.53741	1.5764	0.04816	0.52	0.18	0.431
GSE45365_CD8A_DC_VS_CD11B_DC_IFNAR_KO_MCMV_INFECTION_UP	C7	17	0.50011	1.4465	0.04861	0.588	0.282	0.425
MYC_UP.V1_UP	C6	24	0.48814	1.6908	0	0.667	0.392	0.409
ESC_V6.5_UP_EARLY.V1_UP	C6	24	0.45211	1.6871	0.04251	0.583	0.382	0.364
KIM_RESPONSE_TO_TSA_AND_DECITABINE_UP	C2	17	0.42292	1.462	0	0.471	0.252	0.354
PASINI_SUZ12_TARGETS_UP	C2	15	0.42068	1.4733	0.03937	0.467	0.276	0.34
GO_RESPONSE_TO_BMP	C5	19	0.46386	1.671	0.01047	0.316	0.0963	0.287
GO_CELLULAR_COMPONENT_ASSEMBLY_INVOLVED_IN_MORPHOGENESIS	C5	16	0.52385	1.7083	0.01244	0.625	0.311	0.433
GO_STEM_CELL_DIFFERENTIATION	C5	37	0.49366	1.711	0.01877	0.459	0.268	0.341
GO_REGULATION_OF_STEM_CELL_DIFFERENTIATION	C5	21	0.57195	1.7104	0.03081	0.524	0.268	0.386
GO_NEGATIVE_REGULATION_OF_WNT_SIGNALING_PATHWAY	C5	29	0.38574	1.3922	0.04762	0.276	0.136	0.241

Supplementary Figure Legends

Supplementary Figure S1. Schematic representation of the process used to select the HER2- and HER2+ breast cancer patient datasets.

Supplementary Figure S2. (**A-D**) Principal component analysis (PCA) was performed to determine batch effects among triplicate samples of CCD841 (**A**), CCD33 (**B**), HCT116 (**C**), and HT29 (**D**).

Supplementary Figure S3. (**A-D**) Volcano plot of differentially expressed genes between control (empty vector) and *ERBB2* overexpressing CCD841 (**A**), CCD33 (**B**), HCT116 (**C**), and HT29 (**D**) cells from input RNA-seq. Genes that are significantly upregulated and downregulated based on log2 fold change, respectively) in cells overexpressing *ERBB2* are demarcated by green and blue circles, respectively versus those that did not show a significant change in expression as grey circles ($p > 0.05$).

Supplementary Figure S4. Heat map of the differentially expressed genes between control (empty vector) and *ERBB2* overexpressing normal colon cell lines CCD841 (**A**) and CCD33 (**B**).

Supplementary Figure S5. Heat map of the differentially expressed genes between control (empty vector) and *ERBB2* overexpressing CRC cell line HCT116. Shown are all the differentially expressed genes (**A**) and the top 100 differentially expressed genes (**B**).

Supplementary Figure S6. Heat map of the differentially expressed genes between control (empty vector) and *ERBB2* overexpressing CRC cell line HCT116. Shown are all the differentially expressed genes (**A**) and the top 100 differentially expressed genes (**B**).

Supplementary Figure S7. (**A**) Heat map of the differentially expressed genes between *ERBB2* negative and positive CRC patients. (**B**) Relative *ERBB2* expressions in the 3 *ERBB2*- and 11 *ERBB2*+ patient samples as determined by whole transcriptome RNA-seq.

Supplementary Figure S1

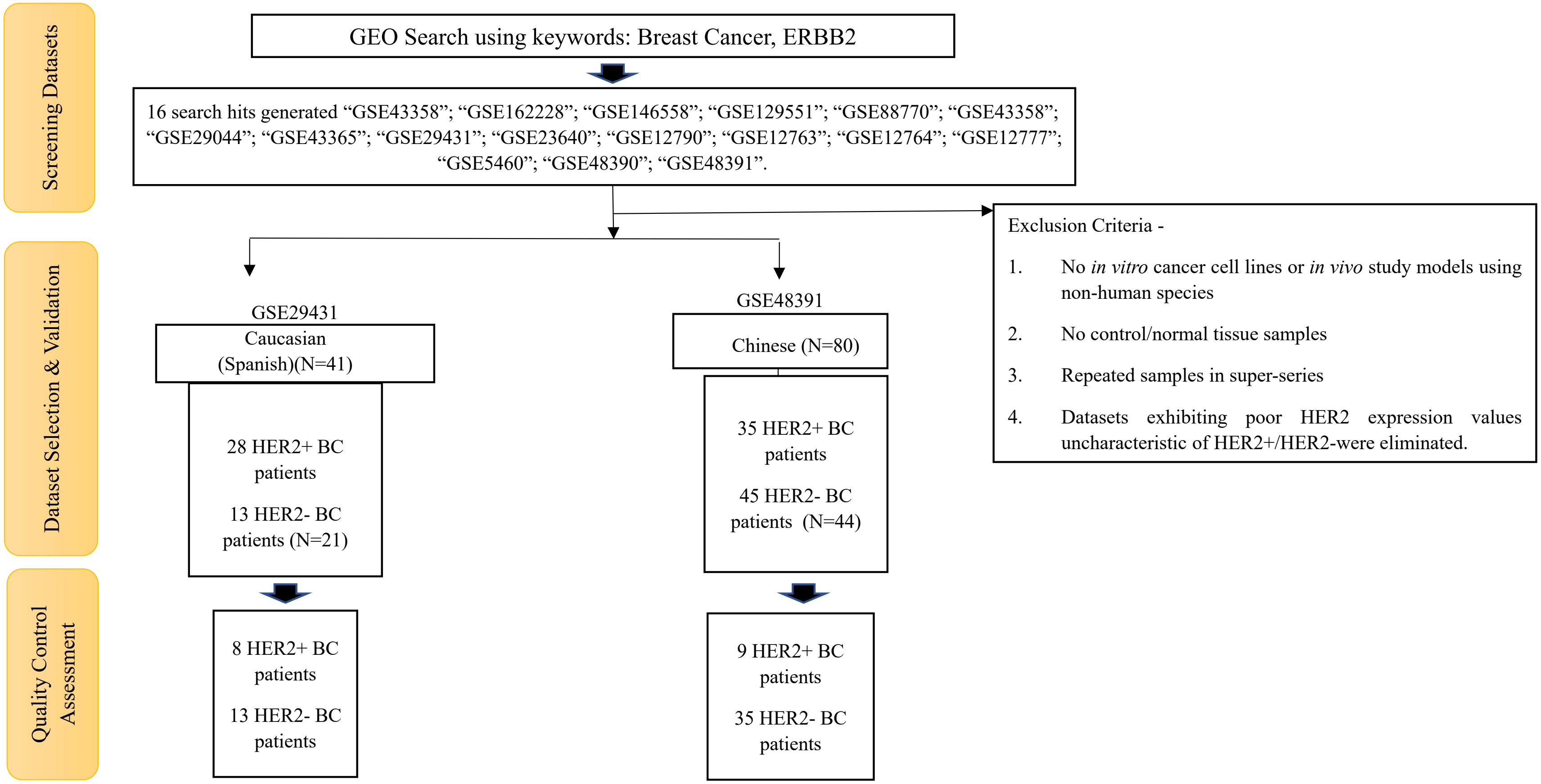

Supplementary Figure S2

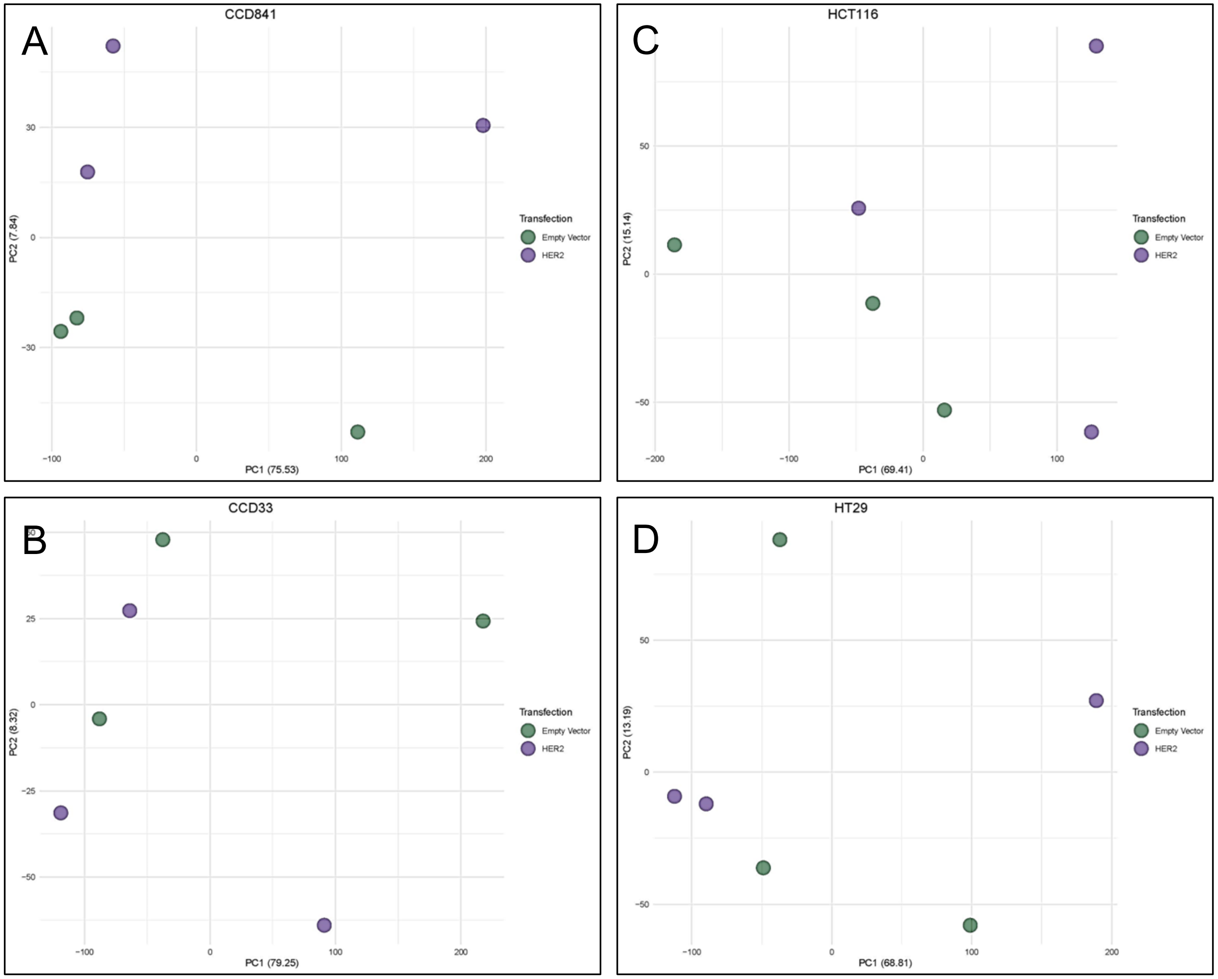

Supplementary Figure S3

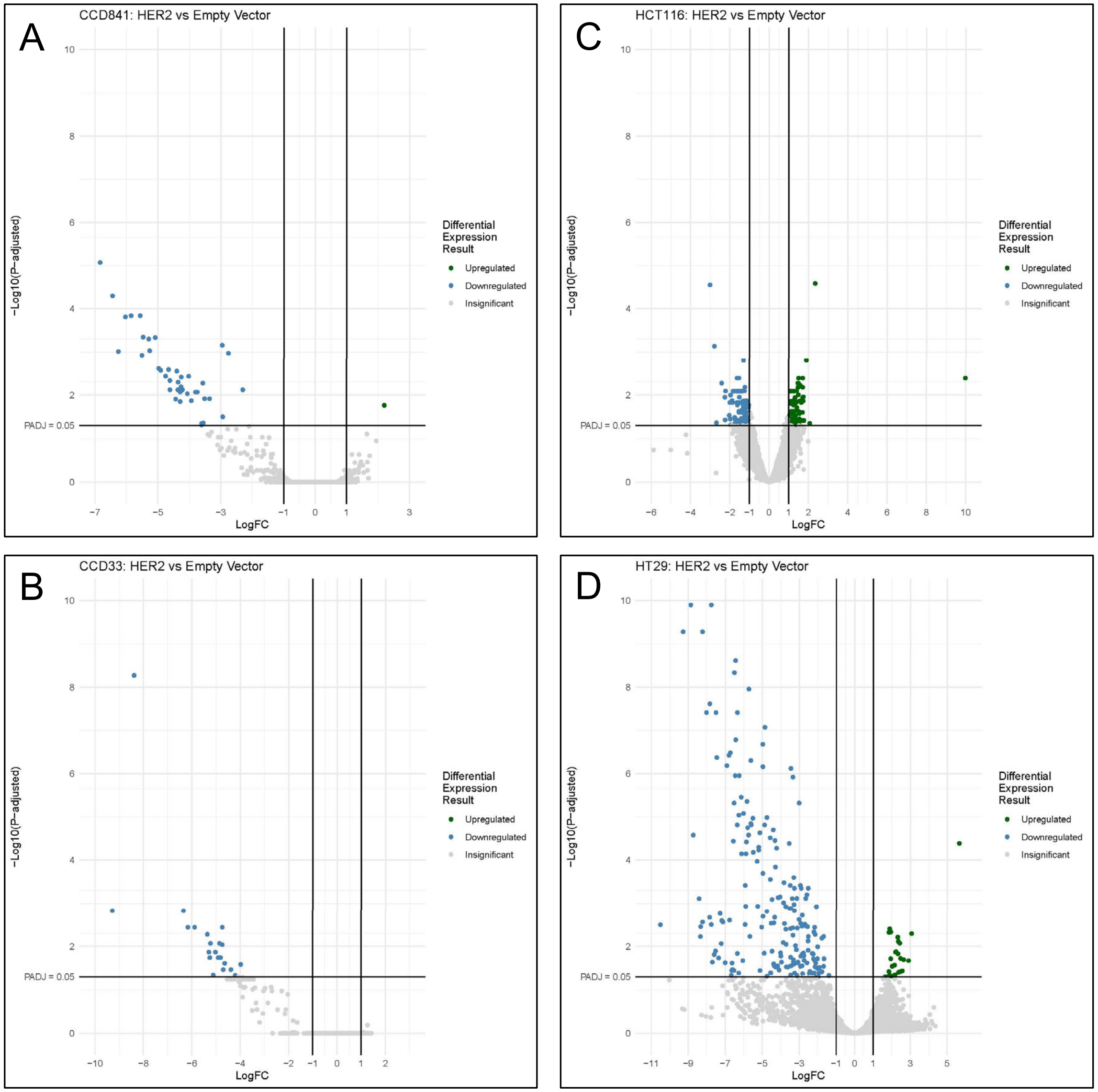

Supplementary Figure S4

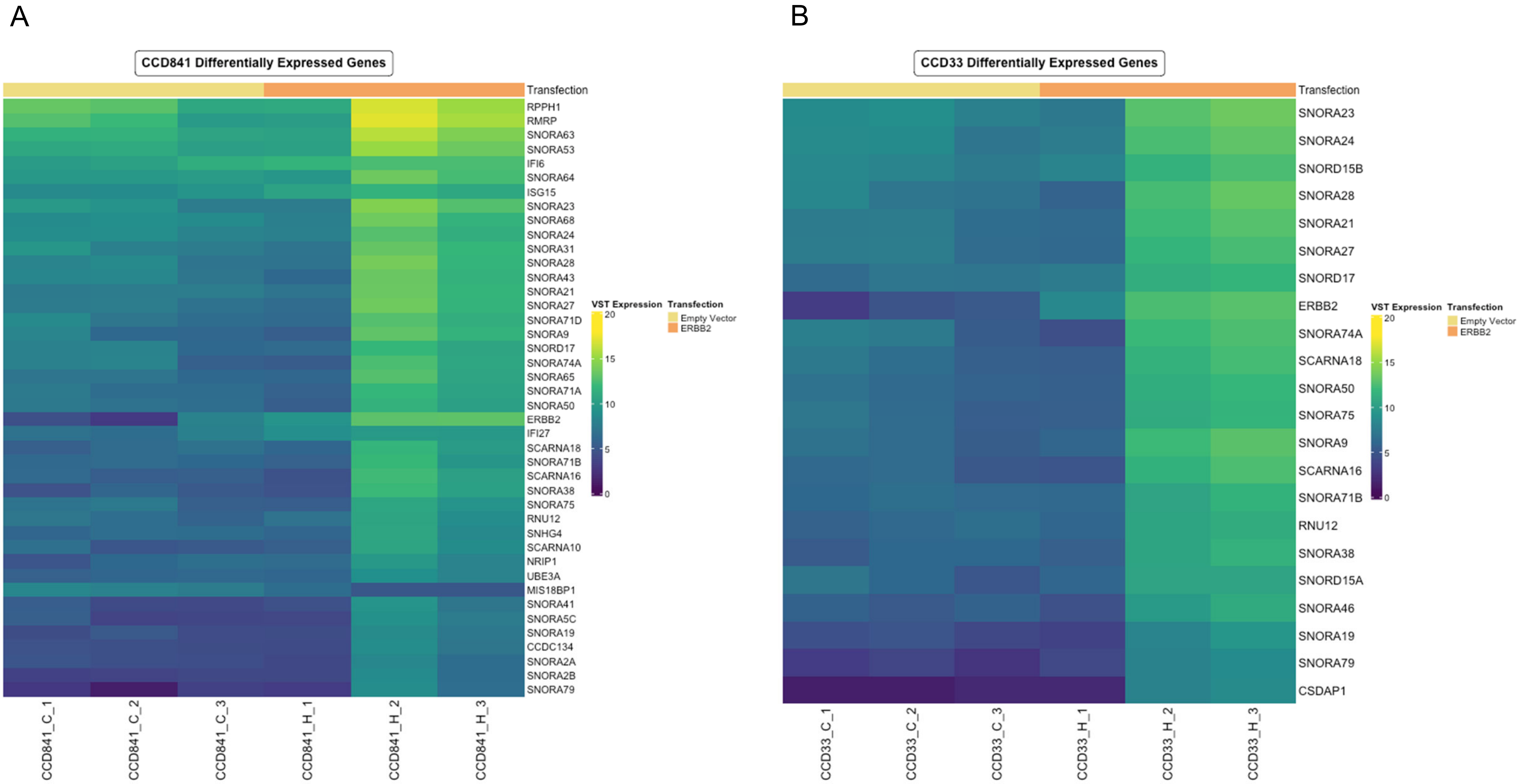

Supplementary Figure S5

A

B

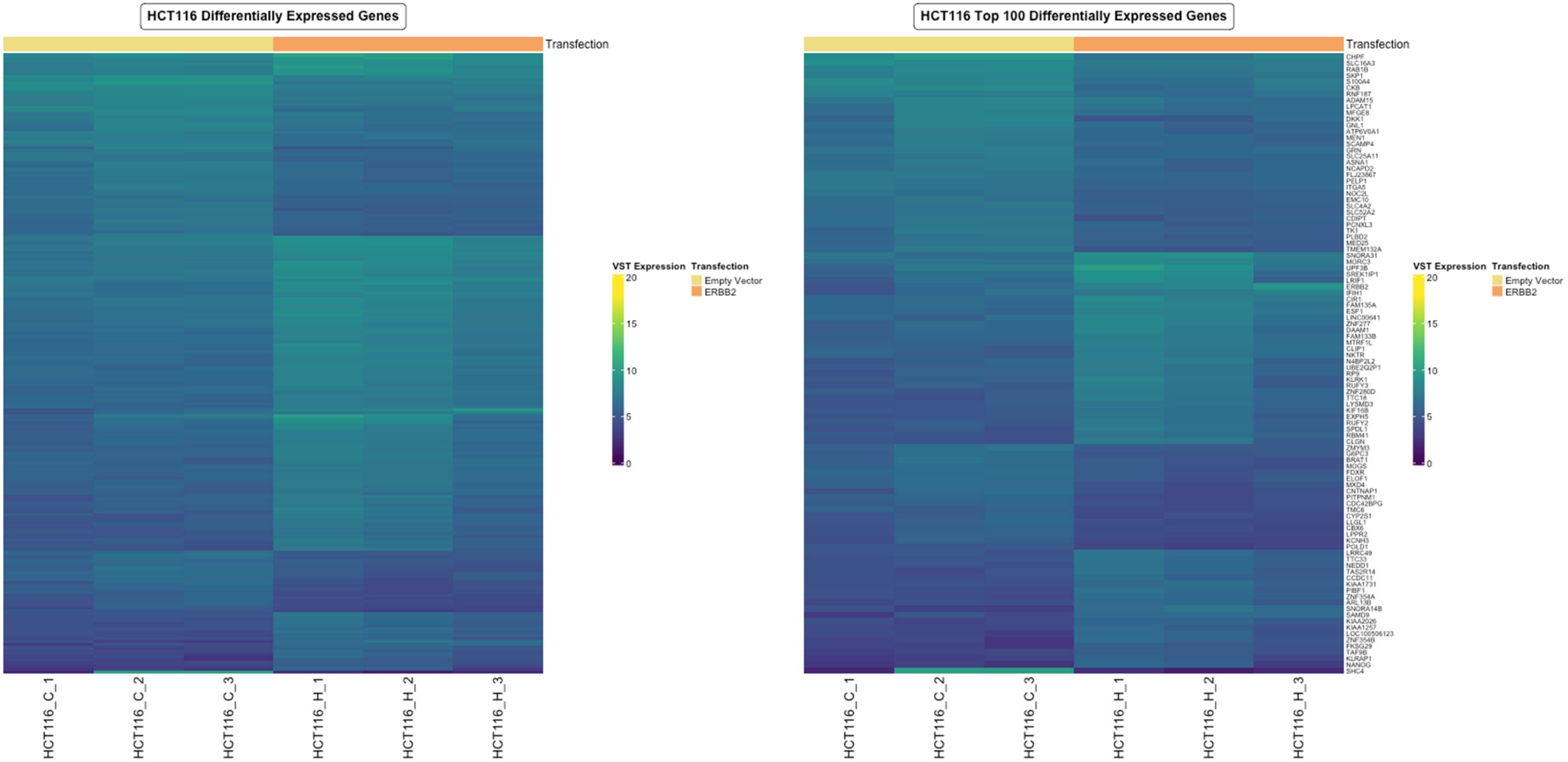

Supplementary Figure S6

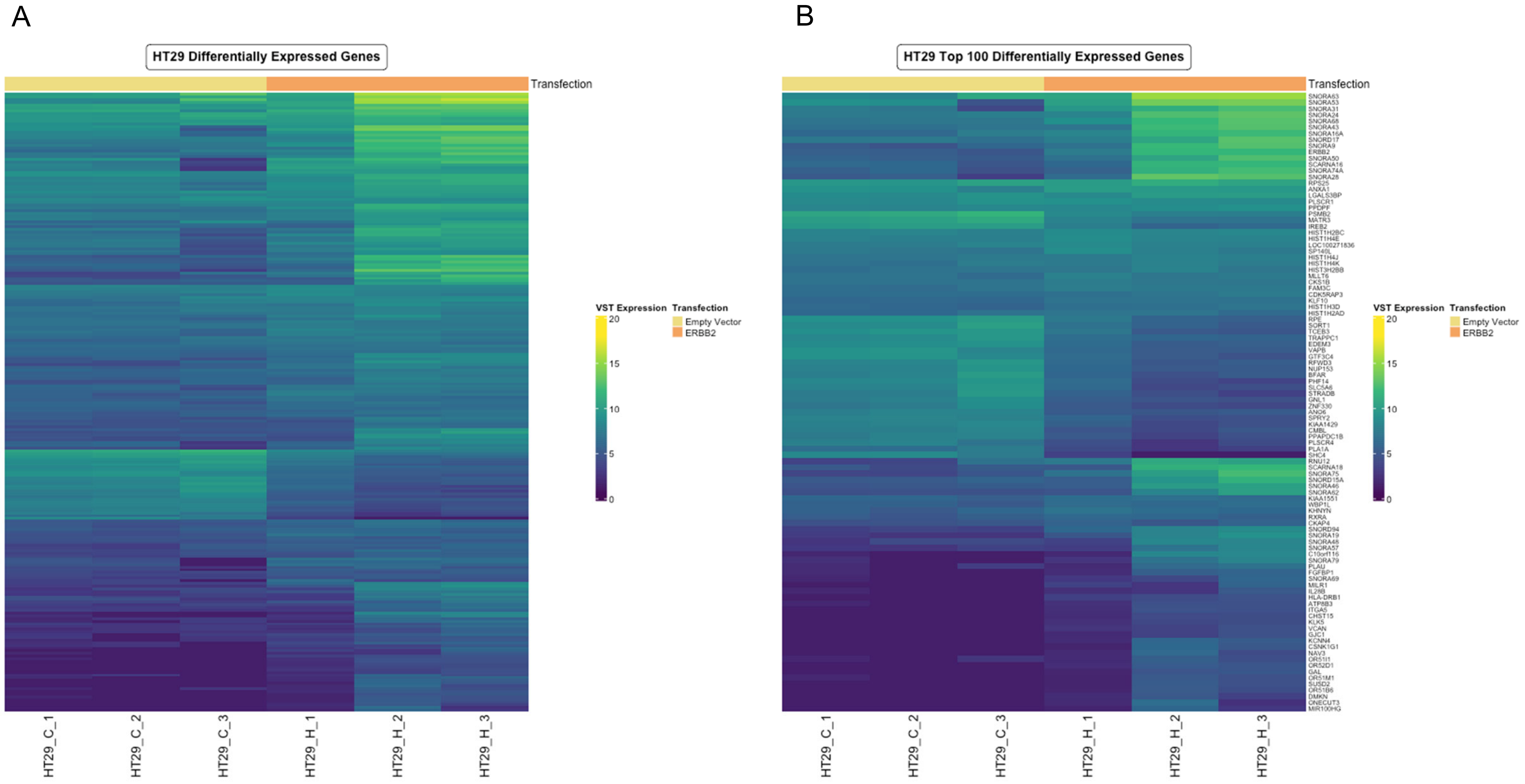

Supplementary Figure S7

A

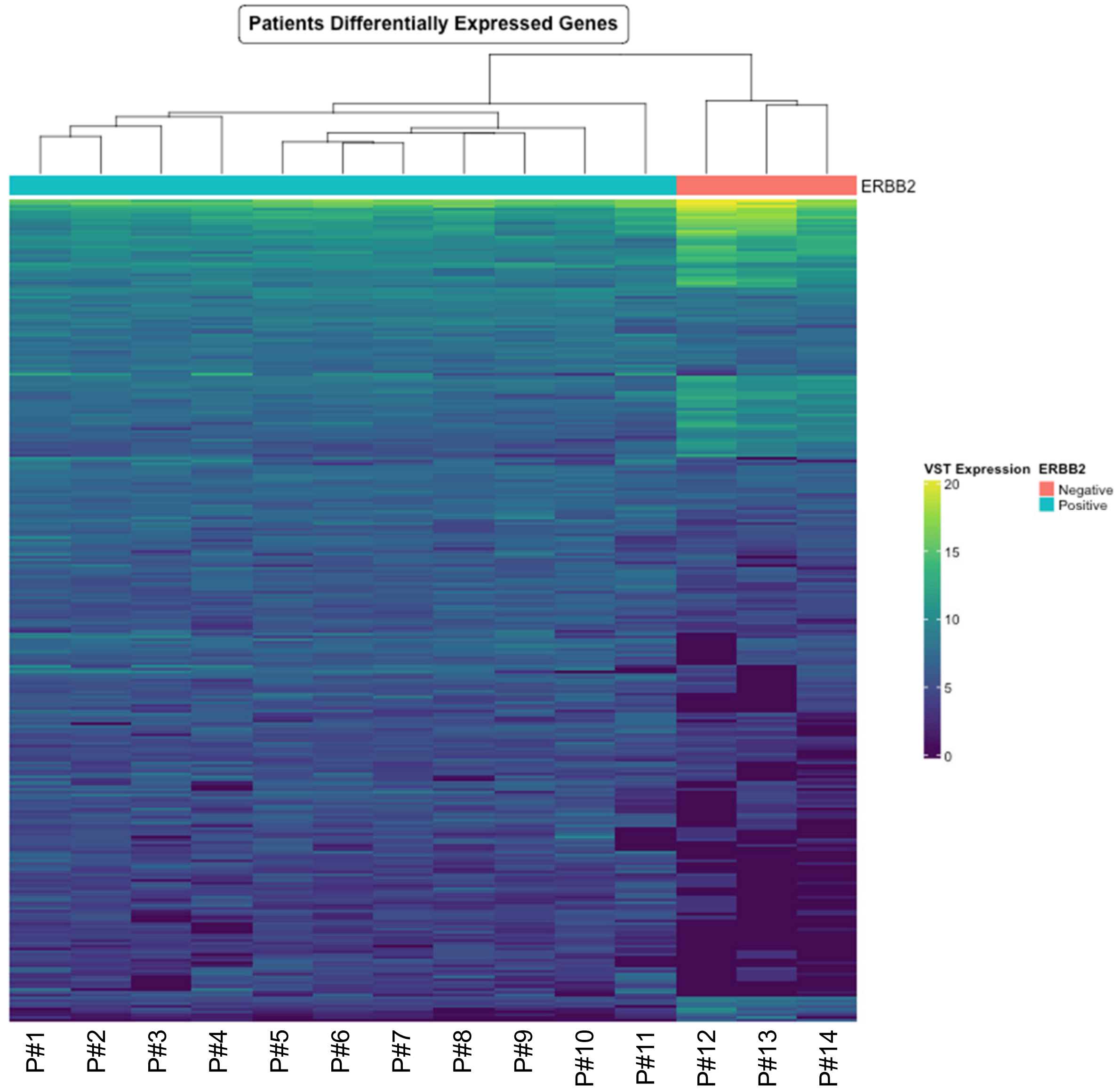

B

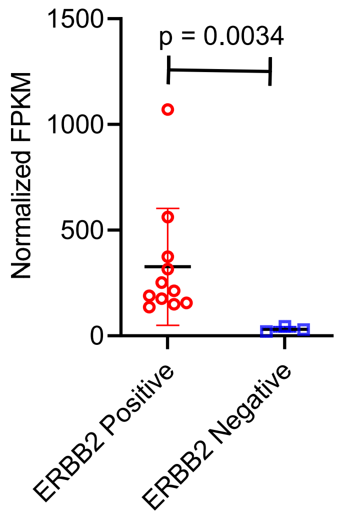

Supplement: Supplementary file 1 [file cancers-15-00130-s001.zip › Supplementary Figures S1-S7 and Tables S5-S7.pdf]
