# Supplementary figures and images for: Transcriptomic Changes Associated with ERBB2 Overexpression in Colorectal Cancer Implicate a Potential Role of the Wnt Signaling Pathway in Tumorigenesis
Source: Cancers (Basel). 2022 Dec 26;15(1):130. doi: 10.3390/cancers15010130 (PMC9817785; doi:10.3390/cancers15010130)

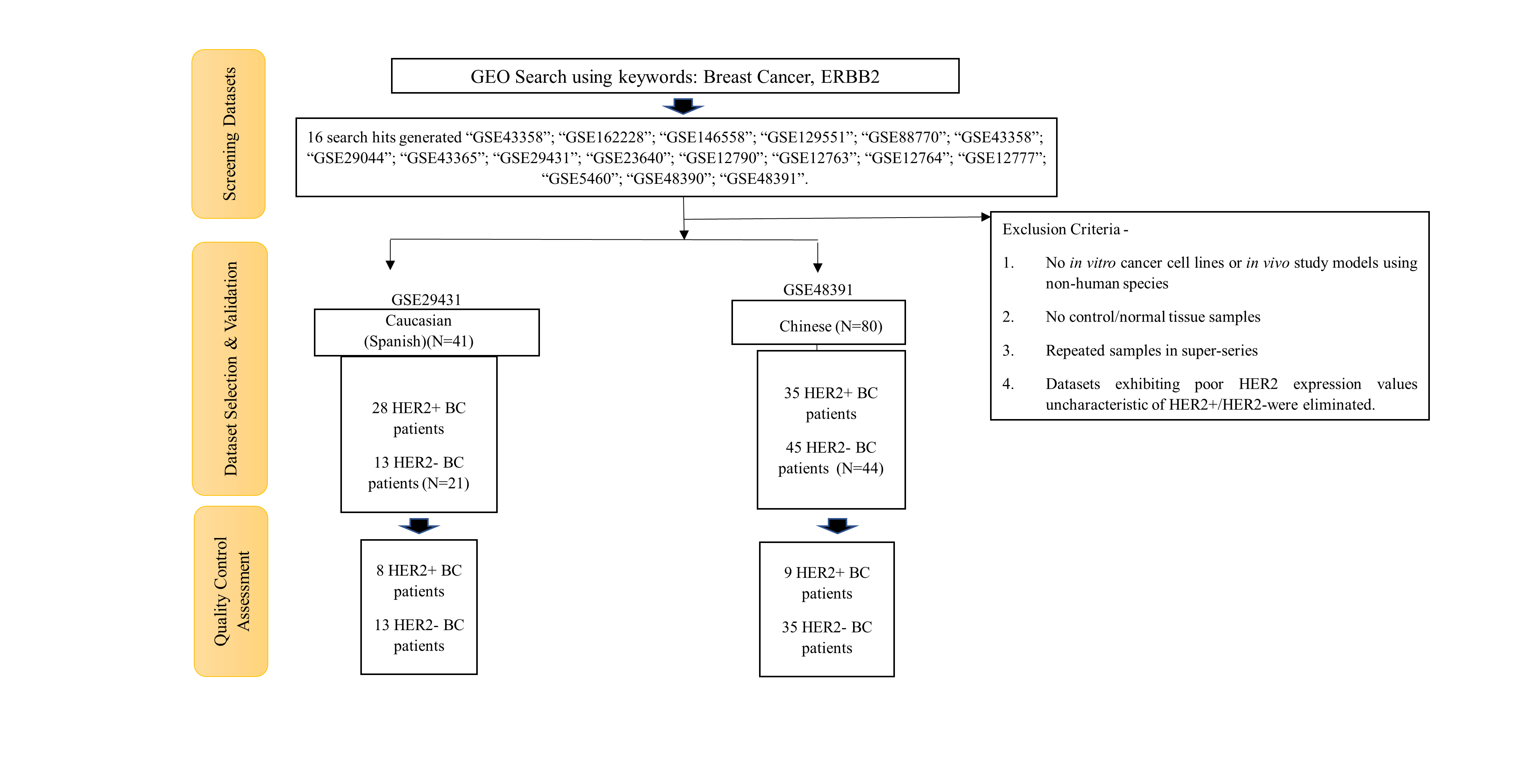

Supplement: Supplementary file 1 [file cancers-15-00130-s001.zip › Figure S1.TIF]

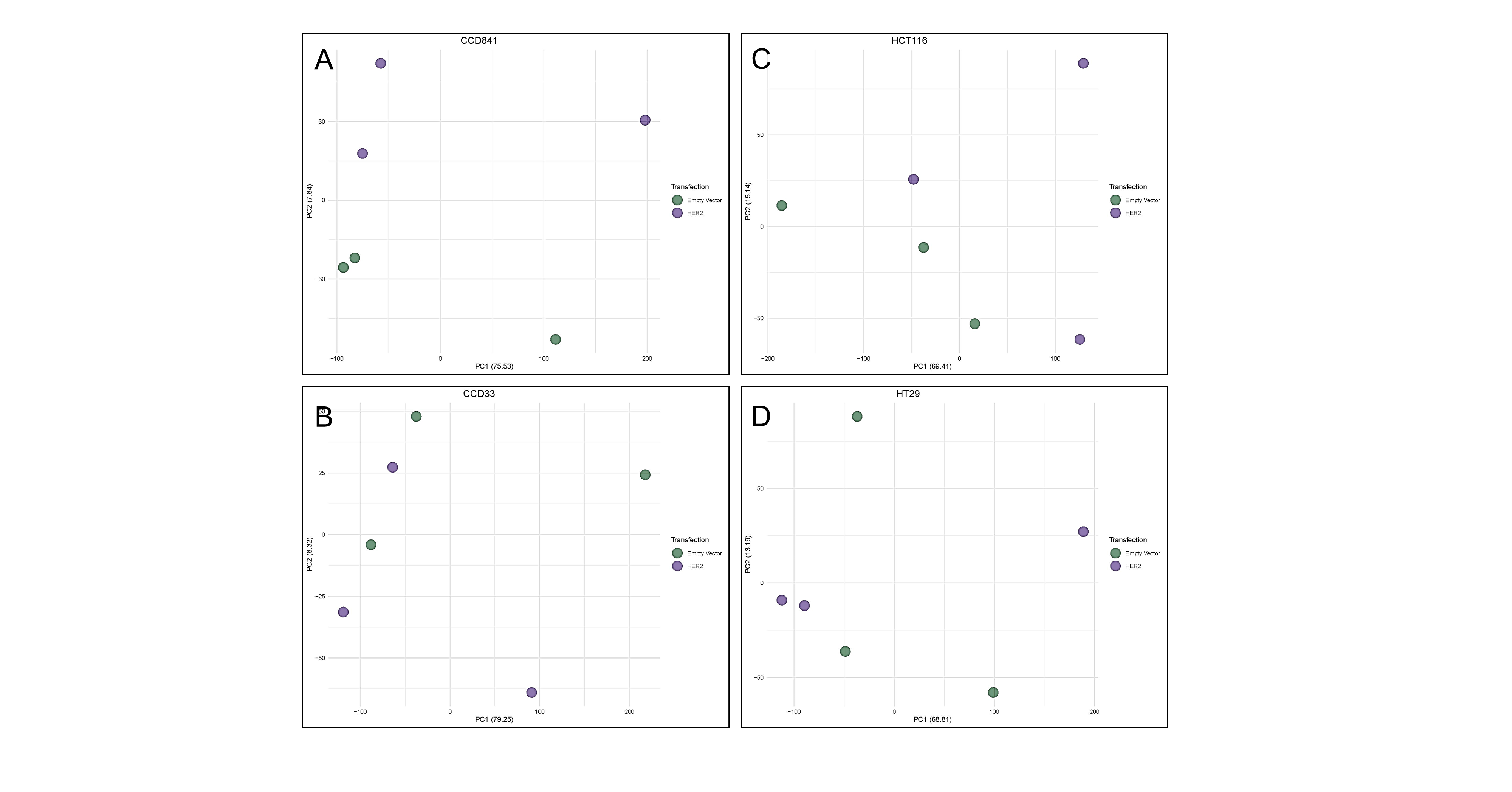

Supplement: Supplementary file 1 [file cancers-15-00130-s001.zip › Figure S2.TIF]

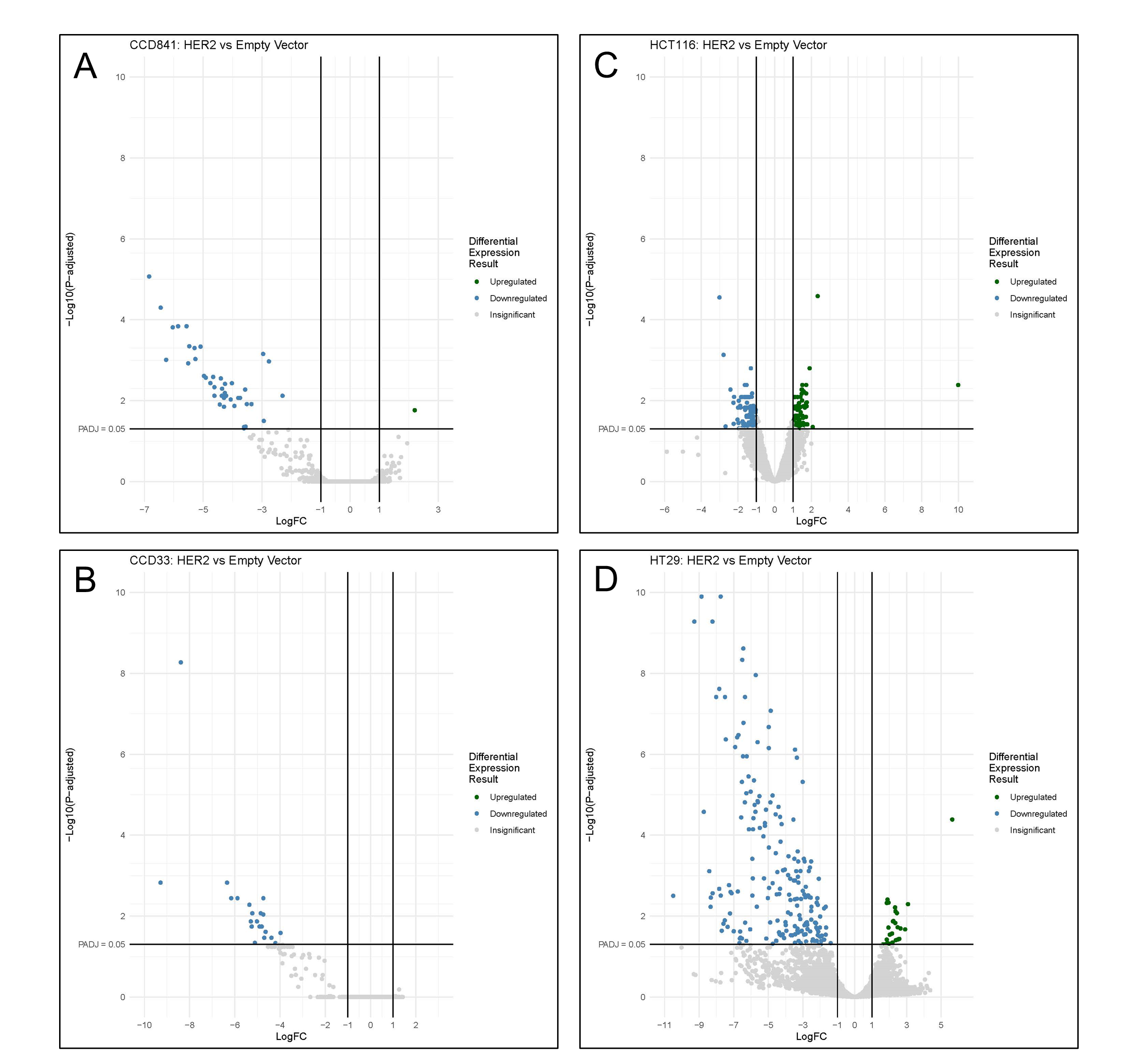

Supplement: Supplementary file 1 [file cancers-15-00130-s001.zip › Figure S3.TIF]

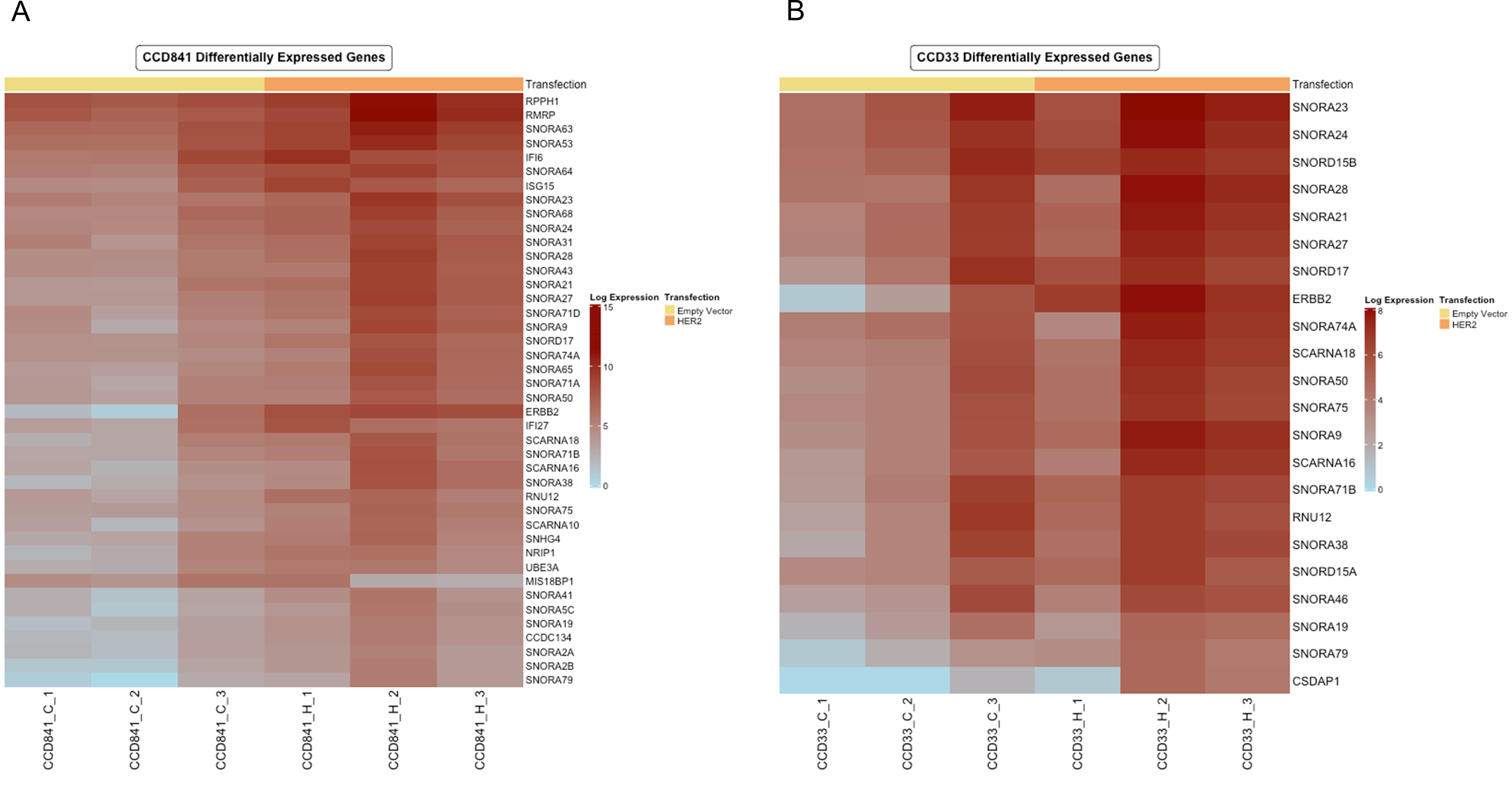

Supplement: Supplementary file 1 [file cancers-15-00130-s001.zip › Figure S4.TIF]

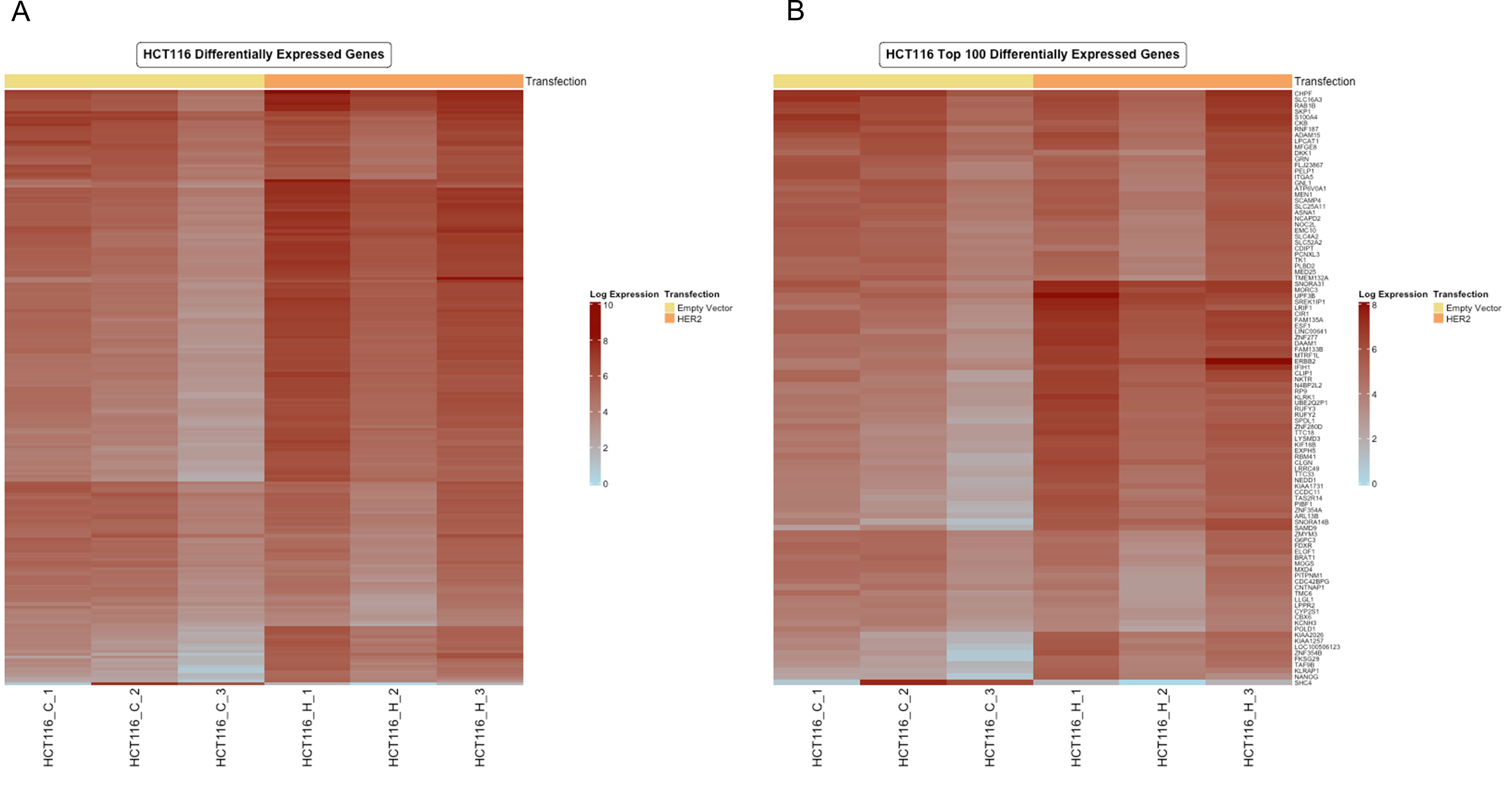

Supplement: Supplementary file 1 [file cancers-15-00130-s001.zip › Figure S5.TIF]

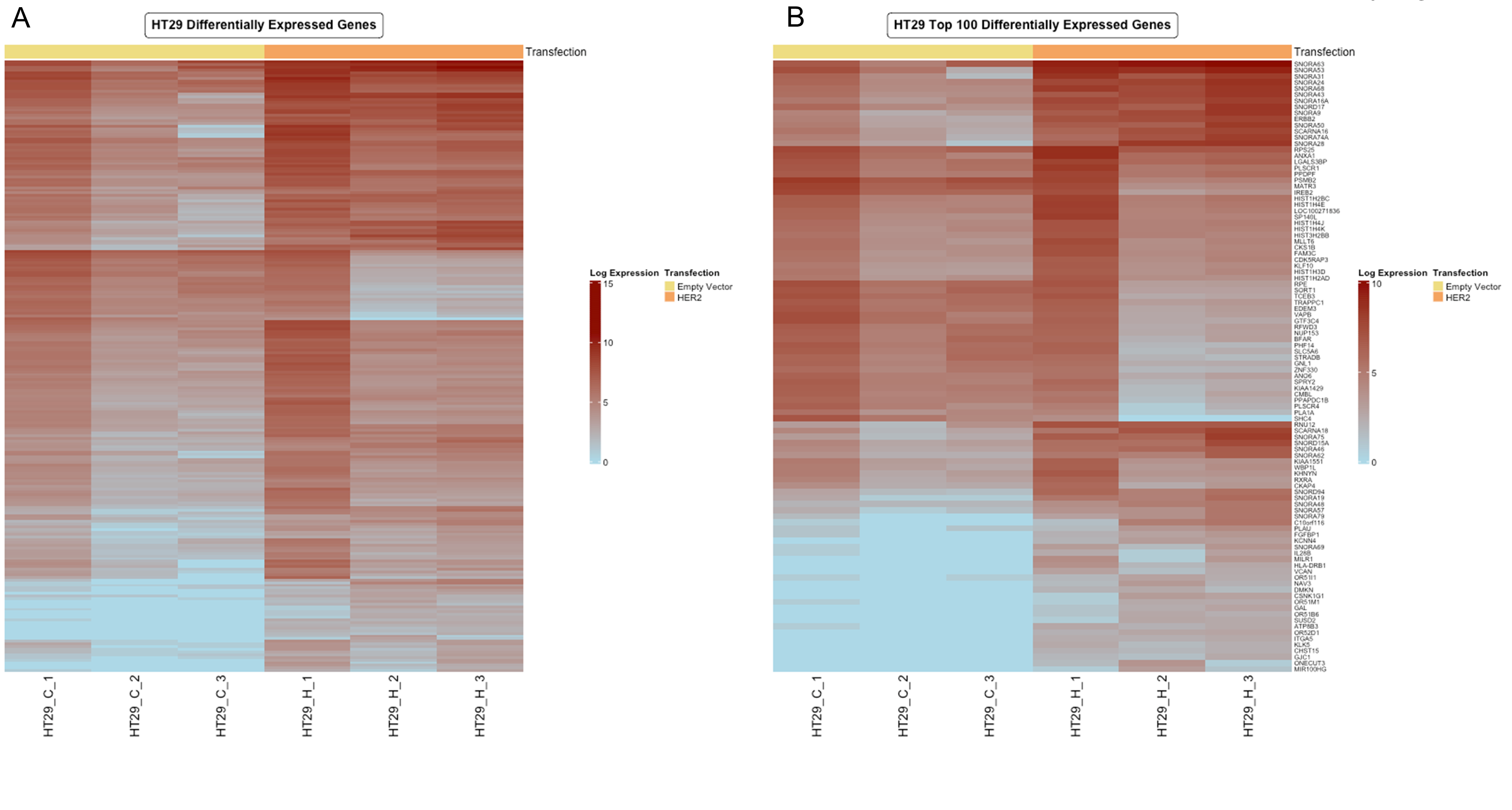

Supplement: Supplementary file 1 [file cancers-15-00130-s001.zip › Figure S6.TIF]

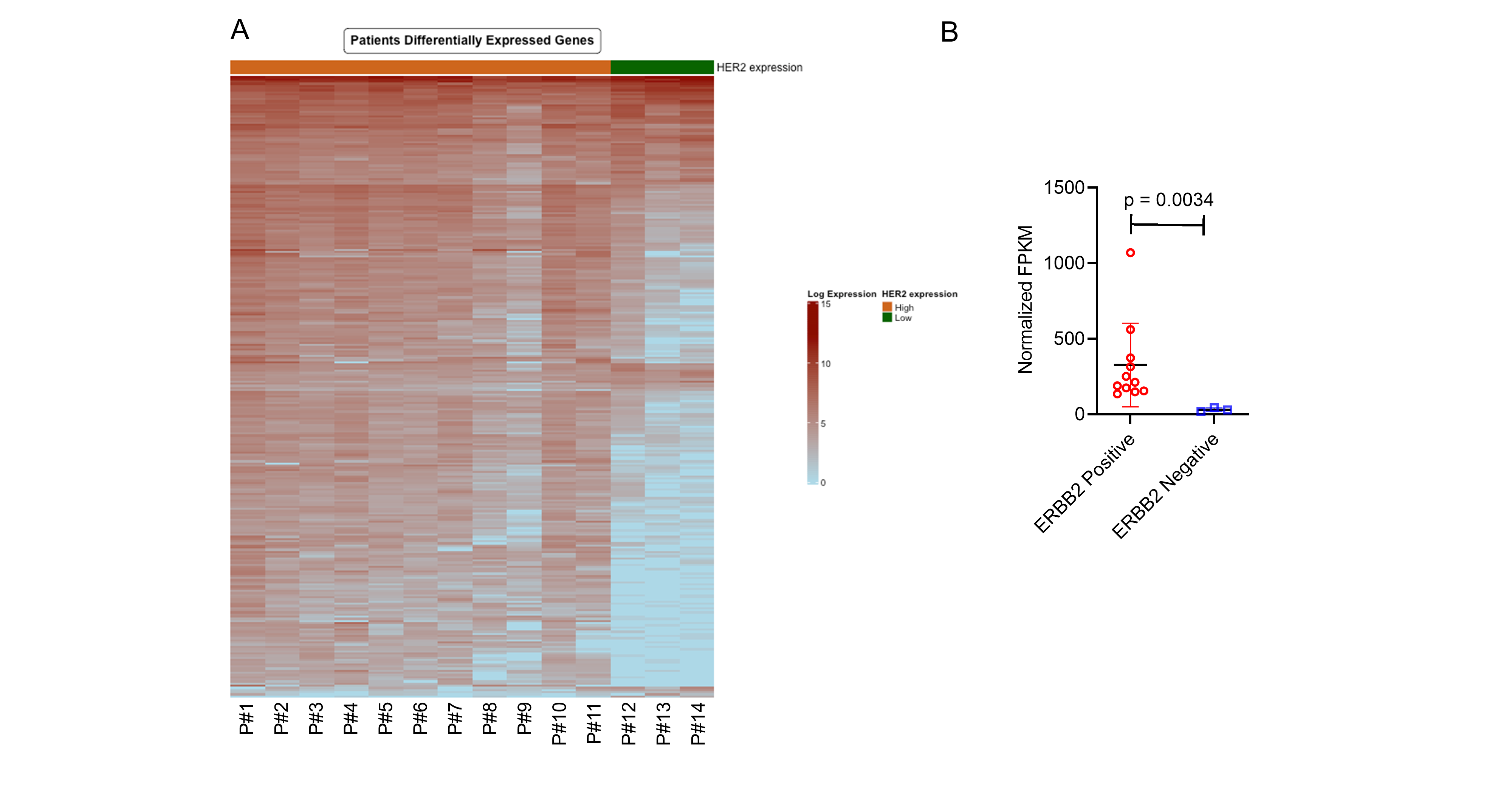

Supplement: Supplementary file 1 [file cancers-15-00130-s001.zip › Figure S7.TIF]
